# Robust registration of SAR and optical images based on deep learning and improved Harris algorithm

**DOI:** 10.1038/s41598-022-09952-w

**Published:** 2022-04-07

**Authors:** Wannan Zhang

**Affiliations:** School of Computer, Central South University, Changsha, China

**Keywords:** Engineering, Mathematics and computing, Optics and photonics

## Abstract

Traditional algorithms can achieve good results when registering homologous images, but it cannot reach satisfying results for registration between synthetic aperture radar (SAR) and optical images. The difficulty is that the image texture information and structures of different modalities is very different which leads to poor registration results. To solve this problem, we present a robust matching framework for registration between SAR and optical images. First, a novel deep learning network is utilized to generate high quality pseudo-optical images from SAR images. Next, feature points are detected and extracted using the multi-scale Harris algorithm. Then the feature points are constructed through the gradient position orientation histogram method. Finally, the actual position of the feature points will be reconstructed through a feedback mechanism for matching. Experimental results demonstrate its superior matching performance with respect to the state-of-the-art methods.

## Introduction

Image registration is a process of aligning two images of the same scene so that corresponding pixels can get the same coordinates^1–5^. This research has been widely used in many practical applications, especially in the field of remote sensing such as change detection, loss assessment, image fusion, and post-disaster rescue. In recent years, with the increasing of high-resolution SAR image data, the registration of SAR and optical images has gradually become a popular topic^6^.

Traditional image registration methods^7,8^ generally include three categories: (1) Feature-based image registration methods, including SIFT-based and SURF-based registration algorithms, etc.; (2): Region-based image registration methods, including MI-based and CCRE-based registration algorithms, etc.; (3) Local structural similarity based methods, including HOPC-based and HIOHC-based algorithms. Although these traditional algorithms can achieve good results when registering homologous images, it cannot achieve satisfying results for registration between SAR and optical images. This is because the image texture information and structures of different modalities are very different which results in poor registration results.

Recently, deep learning (Deep Learning, DL) has begun to emerge in various fields and convolutional neural network (CNN) has been widely used in the area of image processing for its outstanding performance^9^. The amount of CNN-based image processing methods has grown dramatically such as regional convolutional neural network features (Regions with CNN features, R-CNN), region-based fast convolutional neural network (Fast Region-based Convolutional Network, Fast R-CNN), and single-layer multi-frame detectors (Single Shot Multi Box Detector, SSD) and Deep Residual Network (Res Net), etc. These deep learning network models extract and combine different levels of image features^10^. One advantage of this mechanism is that it can realize self-learning of features. Therefore, we introduce the deep learning network for translating SAR images into pseudo-optical images first and then realize registration.

In this paper, we propose a robust matching framework for registration between SAR and optical images. First, a novel deep learning network is utilized to generate high quality pseudo-optical images from SAR images. Next, feature points are detected and extracted using the multi-scale Harris algorithm. Then the feature points are constructed through the GLOH method. Finally, the actual position of the feature points will be reconstructed through a feedback mechanism for matching.

## Methodology

### Proposed network for SAR to optical image translation

In this section, we provide details of the proposed deep learning framework for generating pseudo-optical images from SAR images. The network consists of two main components: colorization network and generative adversarial learning. In the colorization network, we introduce an adversarial loss for better image colorization.

**Figure 1:**
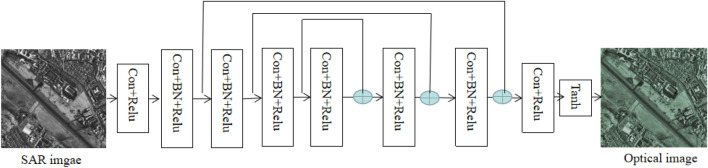
Proposed network for SAR to pseudo-optical image translation.

Deep learning-based image colorization has been studied over the last couple of years^11,12^. Fully leverage the contextual information of an image is the key step during an image colorization neural network for color translation. Generally, an encoder-decoder architecture is added for extracting and utilizing the contextual information. The input image is encoded into a set of feature maps in the middle of the network. But this means that all information flows need pass through all the layers during such a network. Considering the image colorization problem, the sharing of low-level information between the input and output is important since the input and output should share the location of prominent edges. For the above reasons, we add skip connections which is following the general shape of an encoder-decoder CNN as shown in Fig. 1. The colorization sub-network forms a symmetric encoder-decoder with 8 convolution layers and 3 skip connections. For each convolution layer, the kernel size is 3 × 3.

As for the translation of SAR images, one important part is that the output image must be noise free and realistic^13^. One common loss function used in many image translation problems is the L_1_ loss. Although the L_1_ loss has been shown to be very effective for image de-noising problem, it will incentivize an average, grayish color if it is uncertain which of several plausible color values a pixel should take on. In particular, L_1_ will be minimized by choosing the median of the conditional probability density function over possible colors. Thus, the L_1_ loss alone is not suitable for image colorization. Recent studies have shown that the adversarial loss can become aware that gray looking outputs are unrealistic, and encourage matching the true color distribution. Considering the pros and cons of both losses, we combine the per-pixel L_1_ loss and the adversarial loss together with appropriate weights to form our new refined loss function.

### Perform gradient calculation and feature point extraction on the image

The image gradient must be calculated before the feature point extraction. The edge detection Sobel operator can quickly calculate the direction convolution kernel which is required for key point detection of the subsequent Harris algorithm^14^. First define two templates in the horizontal and vertical direction as:

1 $$f_{H} = \left[ {\begin{array}{*{20}c} { - 1} & 0 & 1 \\ { - 2} & 0 & 2 \\ { - 1} & 0 & 1 \\ \end{array} } \right],\;f_{V} = \left[ {\begin{array}{*{20}c} { - 1} & { - 2} & { - 1} \\ 0 & 0 & 0 \\ 1 & 2 & 1 \\ \end{array} } \right]$$

Use the two templates in Eq. (1) to convolve with the image gray value I(x, y) to get the gradient values in the horizontal and vertical directions. Taking into account the scale invariance, the scale parameter α_i_ is introduced, and f_H_ and f_V_.

are regarded as the volume of two rectangular sub-windows and Gaussian kernel functions. The multi-scale Sobel operator used can be expressed as:

2 $${\text{F}}_{{H,\alpha_{i} }} = G{}_{{\alpha_{i} }}*f_{H} ,{\text{F}}_{{V,\alpha_{i} }} = G{}_{{\alpha_{i} }}*f_{V}$$

In the formula, F_H,_ α_i_, F_V_, and α_i_ are the gradients in the horizontal and vertical directions respectively, Gα_i_ is the Gaussian kernel function corresponding to α_i_, and * represents the convolution operation. The scales in the optical image and the SAR image correspond to each other, satisfying;

3 $$\frac{{\alpha_{{{\text{i}} + 1}} }}{{\alpha_{i} }} = k$$

Therefore, the gradient size and direction can be expressed as:

4 $$\left\{ {\begin{array}{*{20}l} {F_{{M,\alpha_{i} }} = \sqrt {(F_{{H,\alpha_{i} }} )^{2} + (F_{{V,\alpha_{i} }} )^{2} } } \hfill \\ {F_{{O,\alpha_{i} }} = \arctan (\frac{{F_{{V,\alpha_{i} }} }}{{F_{{H,\alpha_{i} }} }})} \hfill \\ \end{array} } \right.$$

In the formula, F_M,_α_i_ is the gradient magnitude matrix of the image, and F_O,_α_i_ is the gradient direction matrix.

When the original SIFT algorithm detects the key points of SAR images, the multiplicative speckle noise will have a serious impact on the second derivative used which results in that reliable key points cannot be detected^15^. Therefore, the key point detection method is improved during the SIFT algorithm. Experiments show that the multi-scale Harris detection method can detect key points with higher repeatability and stronger stability, which is better and much faster than the minimum nuclear similarity zone (SUSAN) isocenter detection. Based on the gradient calculation, multi-scale Harris function is used to construct the scale space. The candidate key points of each layer are extracted by calculating the local maximum value, and non-maximum value suppression is performed^16^. The multi-scale Harris function can be expressed as:

5 $$M(\alpha_{i} ) = G_{{\sqrt 2 \alpha_{i} }} *\left[ {\begin{array}{*{20}c} {(G_{{H,\alpha_{i} }} )^{2} } & {(G_{{H,\alpha_{i} }} ) \cdot (G_{{V,\alpha_{i} }} )} \\ {(G_{{V,\alpha_{i} }} ) \cdot (G_{{H,\alpha_{i} }} )} & {(G_{{V,\alpha_{i} }} )^{2} } \\ \end{array} } \right]$$

6 $${\text{R}}(\alpha_{i} ) = \det \left[ {M(\alpha_{i} )} \right] - dtr\left[ {M(\alpha_{i} )} \right]^{2}$$

where: α_i_ is the scale of the image, G_H,_ α_i_, G_V_, and α_i_ are the horizontal and vertical gradients on the scale α_i_ respectively, d is any parameter, det is the value of the matrix determinant, *tr* is the trace of the matrix, and R is the scale space.

### Construct descriptors and perform feature matching

After feature point detection, the GLOH^17^ method is used to establish the descriptor. This descriptor can improve the processing speed of the algorithm while retaining more structural information of the image. It solves the problem of inconsistencies in the main directions of heterogeneous images which is caused by the traditional descriptor creation method, and making the final registration result more stable. At the same time, the nearest neighbor distance ratio (NNDR)^18^ method is used to measure the similarity between descriptors and the FSC (Fast sample consensus) algorithm^19^ is used to delete the wrong matching point pairs.

### Reconstruct feature points of the original image

Considering the problem of image de-redundancy will cause the lack of image pixels and the output image quality is changed when the de-redundant image is directly used for registration, we propose the feature point reconstruction method to make the final registration order and the target of the segment is the original input image^20^. The core idea of feature point reconstruction is that the descriptor is used after de-redundancy to restore the coordinate information in the original image, then compare the deleted elements and coordinate information in set Ω and Ω′ recorded during the de-redundancy process, and calculate the total number of rows and columns removed before the current coordinates. The coordinates of the corresponding points in the original image are the sum of horizontal and vertical coordinates of the feature points in the redundant image, and the number of rows and columns are removed. The process of feature point reconstruction algorithm:

1. Enter the description of the redundant image P = {p_1_, p_2_,…, p_x_}, extract the descriptors of the visible light image and the SAR image;
2. Compare the coordinate information in Θ and Θ′ with the row number and column number recorded in Ω and Ω′ in turn. Take Θ and Ω as an example, the comparison method: arrange all the i_nums_ in Ω in ascending order, and use p_i,1_ in Θ for interpolation sorting. The size of p_i,1_ is the number of rows i_row_ that were removed before that point. The number of columns that were eliminated before the point i_col_.
3. Repeat step 2) for other descriptors to obtain the coordinates in the original image. Taking the ith feature point as an example, the coordinates in the original image are:
  7 $$(q_{i,1} ,q_{i,2} ) = (p_{i + 1} + i_{row} ,p_{i,2} + i_{col} )$$
4. Obtain the position information of feature points of the original image, and perform the parameter estimation of the affine transformation model based on these feature points, then the model is finally to complete the image registration correction.

## Experimental results and analysis

**Figure 2:**
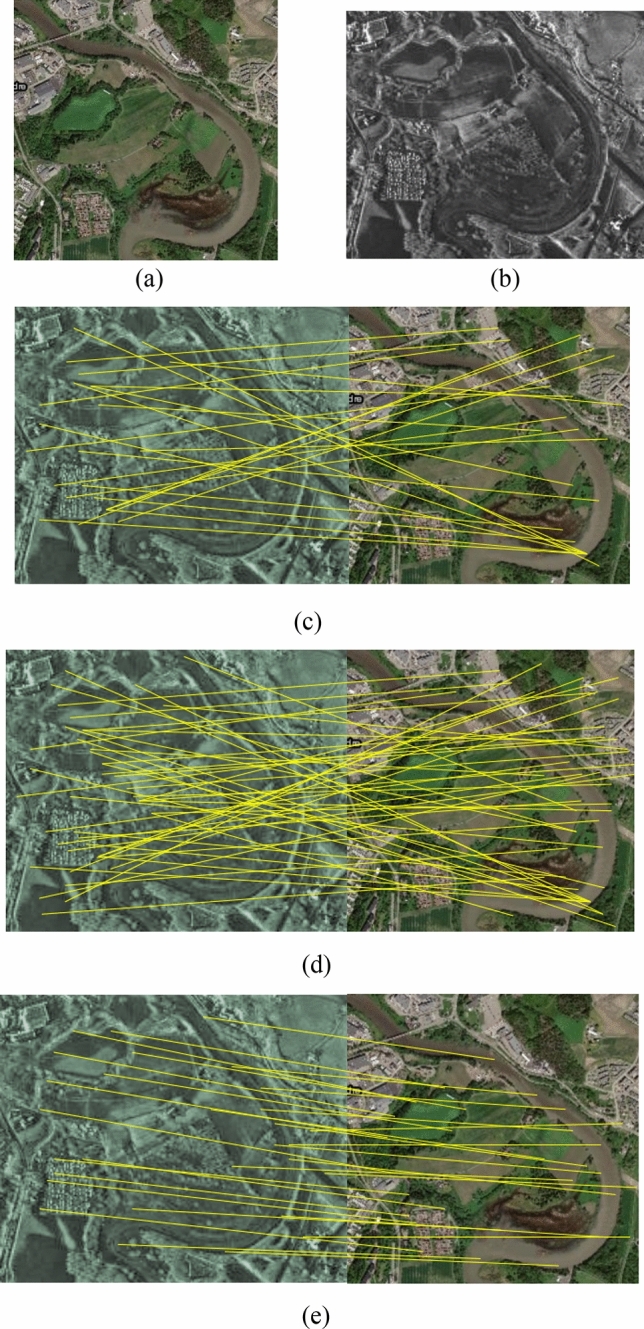
(**a**) Optical image; (**b**) SAR image; Matches found in pair 1 using (**c**) SIFT-M, (**d**) PSO-SIFT, and (**e**) the proposed method.

**Figure 3:**
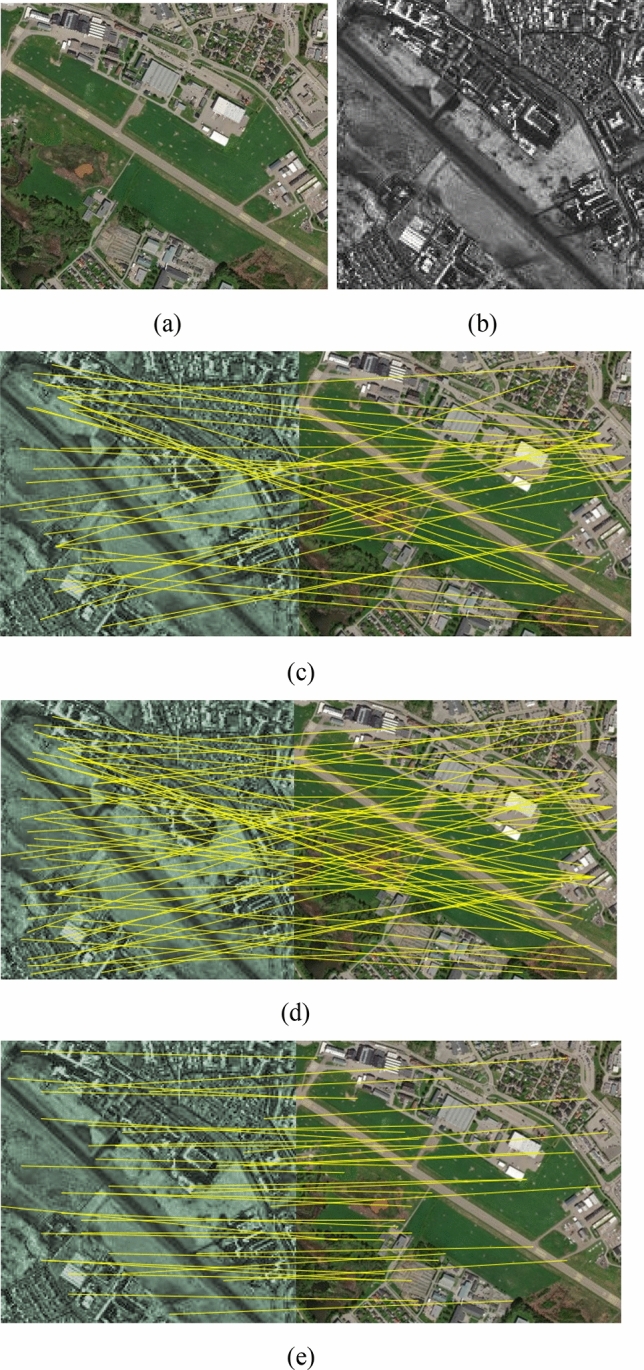
(**a**) Optical image; (**b**) SAR image; Matches found in pair 2 using (**c**) SIFT-M, (**d**) PSO-SIFT, and (**e**) the proposed method.

**Figure 4:**
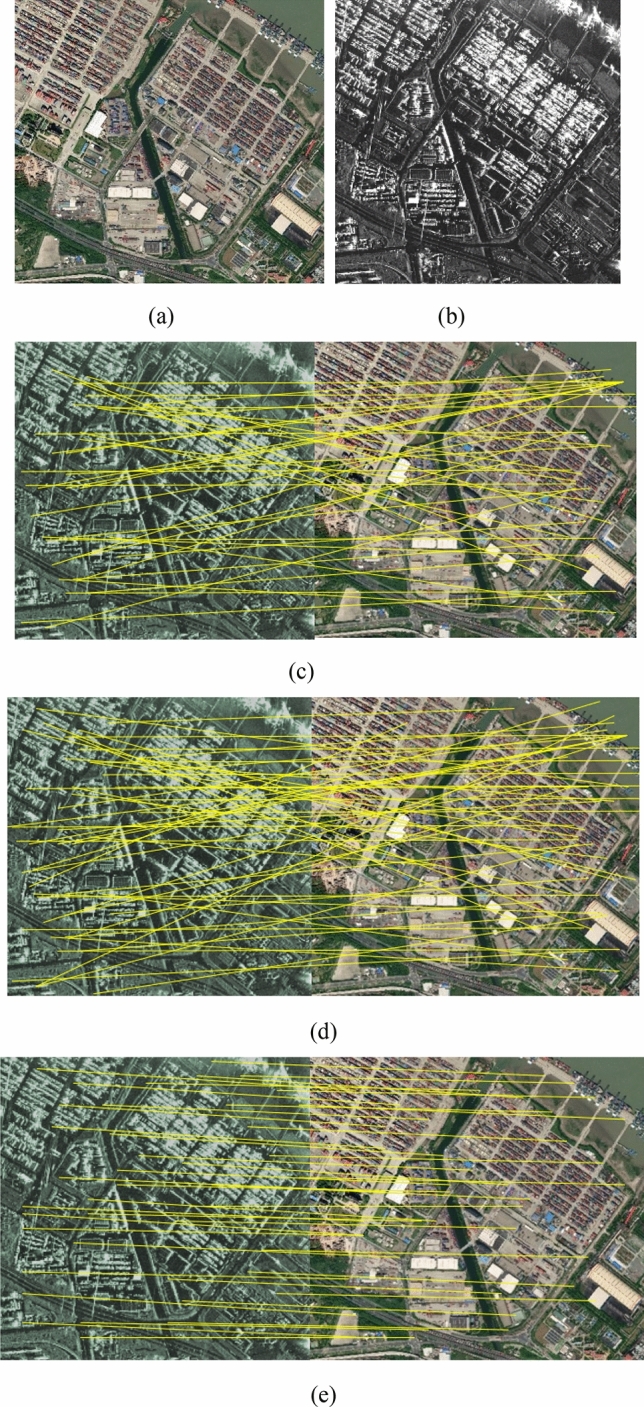
(**a**) Optical image; (**b**) SAR image; Matches found in pair 3 using (**c**) SIFT-M, (**d**) PSO-SIFT, and (**e**) the proposed method.

To evaluate the performance of the proposed method, three pairs of SAR and optical images are experimented. The experiments are compiled with Python3.6, and the network is built through the deep learning framework of Pytorch1.3, and the corresponding CUDA10.0 and cudnn7.0 are configured for GPU acceleration. The test data consists of different characteristics including different resolutions, incidence angles, seasons etc. The dataset description is shown in Table 1. Experimental results are shown in Figs. 2, 3, 4 and Table 2.

**Table 1:** Detailed description of dataset.

Image No	Image source	Size/(pixel × pixel)	Spatial resolution/m	Date	Location
1	TerraSAR-X	580 × 520	2.5	07/2018	Urban area
	Google Earth	580 × 520	3	05/2017	
2	TerraSAR-X	650 × 500	3	12/2010	River area
	Google Earth	650 × 500	3	09/2012	
3	TerraSAR-X	550 × 460	2	10/2018	Suburb area
	Google Earth	550 × 460	3	04/2018

**Table 2:** Quantitative comparison of the proposed method with other SIFT-based algorithms.

Image No	Method	CMR/%	RMSE/pixel	Running Time/s
1	SURF	22.12	3.8331	16.92
	SIFT-M	67.05	1.3782	51.27
	PSO-SIFT	74.96	1.0921	46.73
	Proposed	82.73	0.6014	39.28
2	SURF	25.79	3.4552	17.69
	SIFT-M	78.16	1.4701	53.62
	PSO-SIFT	76.28	1.5714	45.29
	Proposed	85.53	1.0182	39.54
3	SURF	20.56	3.9302	10.72
	SIFT-M	62.29	0.8751	32.49
	PSO-SIFT	53.63	1.0753	25.34
	Proposed	72.98	0.5912	20.18

To quantitatively evaluate the registration performances, we adopt the root-mean-square error (RMSE)^21^ between the corresponding matching keypoints, and it can be expressed as

5 $${\text{RMSE}} = \sqrt {\frac{1}{n}\sum\nolimits_{i = 1}^{n} {\left( {x_{i} - x_{i}^{\prime } } \right)^{2} + \left( {y_{i} - y_{i}^{\prime } } \right)^{2} } }$$

where (x_i_, y_i_) and ($x_{i}^{\prime } ,y_{i}^{\prime }$) are the coordinates of the ith matching keypoint pair; n means the total number of matching points. In addition, correct matching ratio (CMR) is another effective measure which is defined as:

6 $$CMR = \frac{correct\,Matches}{{correspondences}}$$

“correspondences” is the number of matches after using PROSAC, “correctMatches” is the number of correct matches after removing false ones. The results of quantitative evaluation for each method are listed in Table 2.

It can be seen from Table 2 that the SIFT algorithm fails to match in heterogeneous image registration, and the correct matching rate obtained by the SIFT-M^19^ and PSO-SIFT^20^ algorithms is relatively low, and the PSO-SIFT algorithm runs relatively fast. After a certain rule of de-redundancy of the image, the number of feature point pairs for registration can be greatly reduced. The original image reconstruction of the feature point pairs before the affine transformation model estimation can ensure the accuracy of heterogeneous image registration. Therefore, the proposed algorithm reduces greatly the running time as well as improves the efficiency of SAR and optical image registration.

## Conclusion

In this paper, we present a robust matching framework for registration between SAR and optical images. First, a novel deep learning network is utilized to generate high quality pseudo-optical images from SAR images. Next, feature points are detected and extracted using the multi-scale Harris algorithm. Then the feature points are constructed through the GLOH method. Finally, the actual position of the feature points will be reconstructed through a feedback mechanism for matching. Experimental results demonstrate its superior matching performance with respect to the state-of-the-art methods. Future work will mainly comprise a CNN-based framework for learning to identify corresponding patches in SAR and optical images in a fully automatic manner.
